# Effects of the modified field test on exercise-induced peripheral fatigue in non-elite badminton players

**DOI:** 10.3389/fphys.2026.1734224

**Published:** 2026-02-09

**Authors:** Heping Huang, Jian Song, Huiming Huang, Yufan Zeng, Xingchang Li, Su Liu

**Affiliations:** 1 Institute of Physical Education and Sports, Huzhou University, Huzhou, Zhejiang, China; 2 College of Physical Education and Health, China Pharmaceutical University, Nanjing, Jiangsu, China; 3 Faculty of Sports Science, Ningbo University, Ningbo, Zhejiang, China; 4 Institute of Physical Education, SuQian University, SuQian, Jiangsu, China

**Keywords:** badminton, field test, peripheral fatigue, training load, vertical jump

## Abstract

**Objectives:**

This study aimed to examine the effects of a modified badminton field test (FT) on exercise-induced peripheral fatigue and its underlying responses in non-elite male college badminton players. We hypothesized that the repeated high-intensity intermittent efforts during the modified FT would lead to significant reductions in lower-limb muscle performance and elevate markers of peripheral fatigue, including blood lactate accumulation, perceived exertion, and heart rate elevation.

**Methods:**

In a single-arm repeated-measures design, 15 healthy male collegiate badminton players (age: 20.2 ± 0.9 years; BMI: 20.9 ± 1.5 kg/m^2^; playing experience: 1.3 ± 0.4 years) performed five consecutive sets of the field test interspersed with 60 s of passive recovery. Each set involved on-court shuttle runs to eight LED targets and was terminated upon volitional exhaustion, achievement of heart rate ≥92% HRmax, or an RPE ≥18 (Borg 6–20 scale). Markers of peripheral fatigue—vertical jump height (VJ), heart rate (HR), rating of perceived exertion (RPE), and fingertip blood lactate (BL)—were assessed before the test and after each set. Data were analyzed using one-way repeated-measures ANOVA (for VJ, HR, RPE) and paired t-tests (for BL).

**Results:**

VJ height progressively decreased from 41.8 ± 4.7 cm at baseline to 25.5 ± 4.5 cm after set 5 (Δ = 39.9%, η^2^p = 0.60, large effect size; p < 0.001). Concurrent increases were observed in HR (63.5 ± 3.8 to 178.0 ± 3.9 bpm; η^2^p = 0.96, large effect size), RPE (6–18.7 ± 0.9; η^2^p = 0.95, large effect size), and BL (2.82 ± 1.12 to 16.07 ± 2.52 mmol L^-1^; Cohen’s d = 6.8, large effect size; all p < 0.001). These convergent metabolic, neuromuscular, and perceptual responses confirm the induction of pronounced peripheral fatigue.

**Conclusion:**

A single 15-min modified badminton FT reliably elicits marked peripheral fatigue in non-elite male players. The protocol provides coaches with an ecologically valid, low-cost and high-safety tool to monitor training load and mitigate fatigue-related injury risk. Future research should validate the FT in female and youth cohorts and explore longitudinal applications.

## 1 Introduction

Exercise-induced peripheral fatigue—defined as a transient reduction in skeletal muscle force-generating capacity—is a multifactorial phenomenon that limits athletic performance and increases injury risk in racquet sports (Allen et al., 2008; Enoka and Duchateau, 2008). Badminton is characterized by repeated explosive movements, rapid changes in direction, and short high-intensity rallies interspersed with brief recovery periods (Cabello-Manrique and González-Badillo, 2003). These demands induce considerable metabolic and neuromuscular stress, which manifests as decreased vertical jump (VJ) height, elevated blood lactate concentration (BL), increased rating of perceived exertion (RPE), and autonomic imbalance (Cabello-Manrique and González-Badillo, 2003; Fernández-Fernández et al., 2009; Girard et al., 2007). Although peripheral fatigue has been widely studied in elite badminton players (Abian-Vicén et al., 2012; Phomsoupha and Laffaye, 2015), evidence in non-elite populations—particularly university-level athletes—remains limited.

Badminton is one of the most popular racquet sports in the world. The results of epidemiological studies have demonstrated the incidence of injuries in badminton to be 2.9 injuries/player/1000 badminton hours and almost one-third of all injuries included from strain and sprains of the lower extremities. The results of a biomechanical study demonstrated that peripheral fatigue induces ankle kinematic and lower leg muscle activity changes which may increase the risk of ankle sprain in badminton players (Herbaut and Delannoy, 2020). At present, there is a lack of validated, low-cost, and sport-specific tools to quantify peripheral fatigue in non-elite badminton players. Traditional laboratory-based assessments—including isokinetic dynamometry, magnetic resonance spectroscopy, and maximal voluntary contraction with twitch interpolation—offer precise mechanistic insights but are costly, time-consuming, and ecologically limited (Kellis and Baltzopoulos, 1995; Place et al., 2010). Existing field tests such as the Yo-Yo Intermittent Recovery Test were originally designed for soccer or basketball and do not adequately replicate the intermittent, multidirectional movement patterns specific to badminton (Krustrup et al., 2003; Castagna et al., 2006). As a result, these tests tend to underestimate sport-specific fatigue and have limited practical value for coaches outside elite training environments.

To address these issues, we developed a modified badminton-specific field test (FT) that incorporates on-court shuttle running, lunging, and striking actions to better simulate match-play demands (Chin et al., 1995). The primary aim of this study was to evaluate whether the modified FT can reliably induce peripheral fatigue in non-elite male collegiate athletes and provide a quantitative assessment of metabolic, neuromuscular, and perceptual responses, together with an evaluation of its safety. We hypothesized that participation in the FT would lead to (i) a significant reduction in lower-limb neuromuscular performance, as indicated by decreased VJ height, and (ii) concurrent increases in BL, RPE, and heart rate (HR). A secondary aim was to assess the practical utility of the FT as a low-cost, time-efficient tool for monitoring training load and reducing fatigue-related injury risk in resource-constrained settings.

## 2 Materials and methods

### 2.1 Study design

This study was a single-arm, non-randomized controlled trial, which followed the Transparent Reporting of Evaluations with Nonrandomized Designs (TREND) guidelines (Des Jarlais et al., 2004). To enhance the completeness and transparency of reporting, target values derived from the literature were used for the assessment of peripheral fatigue. The modified badminton field test was designed in accordance with the field-based fatigue research framework proposed by Ooi and Tan (2023); to ensure ecological validity for non-elite badminton players.

### 2.2 Participants

**FIGURE 1:**
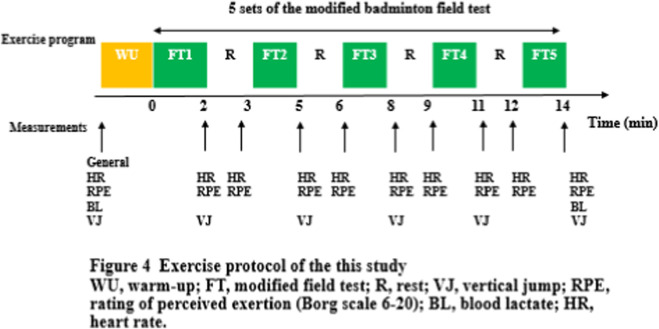
The protocol of the study using the modified badminton field test.

Non-elite male collegiate badminton players were recruited through the university’s WeChat platform. Testing was conducted at the laboratory of the Faculty of Sport Science, Gannan Normal University, China. The inclusion criteria followed the pre-exercise screening guidelines established by Phomsoupha and Laffaye (2024a): (i) age 18–25 years; (ii) regular badminton training at least twice per week during the preceding 6 months; and (iii) not suffering from of musculoskeletal, cardiovascular or metabolic disease as confirmed by the Adult Pre-Exercise Screening System (APSS) (ACSM, 2022). Exclusion criteria comprised current injuries, use of performance-enhancing substances, or long-term medication that could potentially modulate fatigue responses. Among the recruited volunteers, two were excluded—one due to an ankle sprain and another who declined to provide informed consent. Ultimately, a final cohort of 15 male participants (age 20.2 ± 0.9 years; BMI 20.9 ± 1.5 kg·m^-2^; training history 1.3 ± 0.4 years) completed all experimental procedures (Figure 1).

### 2.3 Modified badminton FT

**FIGURE 2:**
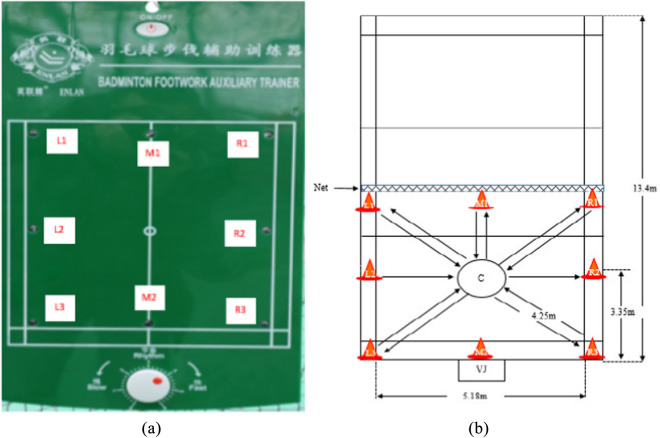
Modified badminton field test (FT): **(a)** badminton footwork auxiliary trainer; **(b)** modified badminton field test with the 8-touched point.

The FT was adapted from the validated 1995 elite-player protocol (Chin et al., 1995) and refined according to the 2022 kinematic recommendations of Claudino et al. (2022) to maximize sport-specificity. The test consisted of five consecutive sets of on-court shuttle running to eight LED targets positioned around a half-court (Figure 2). Each set ended when any of the following criteria were met: (i) volitional exhaustion, (ii) HR ≥ 92% HRmax, or (iii) RPE ≥18 on the Borg 6–20 scale. A 60-s passive recovery was enforced between sets; total testing time averaged 15 min.

### 2.4 Outcome measures

All measurements were conducted in the laboratory of Faculty of Sport Science, Gannan Normal University under standardized conditions (22 °C ± 1 °C, 55% ± 5% relative humidity) between 08:00 and 11:00 to minimize circadian variation.

1. VJ was measured using a contact-mat (Takei 5414, Japan) via the countermovement jump technique; the best of three attempts was recorded for analysis (Claudino, 2021).
2. HR was recorded continuously using a telemetric system (Polar H10, Finland) and expressed as a percentage of the individual’s maximum HR (HRmax), which was determined during an incremental treadmill test conducted 48 h before the experiment (ACSM, 2022).
3. RPE was obtained immediately following each set using the Borg 6–20 scale.
4. Fingertip capillary blood samples (20 µL) were collected pre-test and post-test for LB analysis with a validated portable analyzer (EKF Lactate Scout+, Germany) (Ooi and Tan, 2023).

### 2.5 Statistical analysis

Data normality was confirmed using the Shapiro-Wilk test. One-way repeated-measures analysis of variance (ANOVA) with a time factor, followed by Bonferroni *post hoc* tests, was employed to analyze VJ, HR, and RPE data. Changes in BL levels before and after the test were analyzed using paired-sample t-tests. For the repeated-measures ANOVA, effect sizes were reported as partial eta-squared (η^2^p) and interpreted as follows: small effect (η^2^p ≥ 0.01), medium effect (η^2^p ≥ 0.06), and large effect (η^2^p ≥ 0.14) (Lakens, 2023). For the t-tests, effect sizes were reported as Cohen’s d and interpreted as follows: small effect (d ≥ 0.2), medium effect (d ≥ 0.5), and large effect (d ≥ 0.8). The statistical significance level was set at p < 0.05. All analyses were performed using SPSS version 26.0 (IBM Corp., USA).

Sample Size Estimation: An *a priori* power analysis was conducted using G*Power software. For a one-way repeated-measures ANOVA with five measurements, an alpha level of 0.05, a power (1-β) of 0.80, and an anticipated effect size (η^2^p) ranging from 0.4 to 0.5 (based on effect sizes reported in prior fatigue studies), the analysis indicated a required sample size of 11–14 participants. The final effective sample size in this study met this statistical requirement.

## 3 Results

Safety Assessment: All 15 participants successfully completed the five sets of the modified FT protocol without any incidence of exercise-related injuries or other adverse events (Figures 1, 2). Baseline descriptive data are presented in Table 1. Mean HRmax determined during the incremental treadmill test was 193.9 ± 0.7 bpm, corresponding to 92% HRmax = 178.1 ± 0.5 bpm—values that are consistent with recent badminton-specific cardiopulmonary data (Phomsoupha and Laffaye, 2024b).

**TABLE 1:** The demographic and baseline characteristics of 15 male non-elite badminton players.

Demographic and baseline characteristics	Non-elite badminton players (n = 15)^a^
Age, years	20.2 ± 0.9
Weight, kg	63.2 ± 5.1
Height, cm	173.0 ± 4.1
BMI, kg/m^2^	20.9 ± 1.5
Height with arm reach, cm	219.6 ± 6.6
Blood pressure	
Systolic blood pressure, mmHg Diastolic blood pressure, mmHg	117.8 ± 13.3 58.4 ± 10.7
Experience of badminton playing, year	1.3 ± 0.4
Baseline heart rate. beats/min	63.5 ± 3.8
Max heart rate, beats/min	193.9 ± 0.7
86% of max heart rate, beats/min	166.6 ± 0.5
92% of max heart rate, beats/min	178.1 ± 0.5

^a^ Mean ± SD.

**FIGURE 3:**
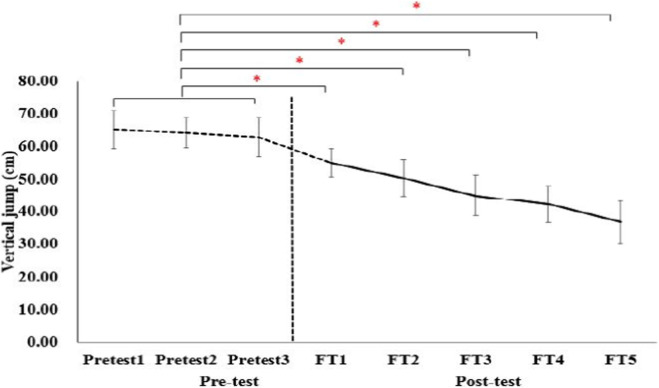
Changes of VJ in pre-test and post-test of each FT. *, significant differences from average of pre-test (p < 0.001).

A significant main effect of set number was observed for VJ height (F_4, 196_ = 72.4, p < 0.001, η^2^p = 0.60, large effect size). Baseline VJ averaged 41.8 ± 4.7 cm and decreased progressively after each set, reaching 25.5 ± 4.5 cm post-set 5 (Figure 3). This result represents a mean reduction of 39.9% ± 6.6% (95% CI 38.1%–41.7%), which is comparable to the 35%–45% decline reported in elite players after simulated singles matches (Ooi and Tan, 2023). Pair-wise comparisons revealed that each subsequent set resulted in a significantly lower VJ than the preceding set (all p < 0.001).

**FIGURE 4:**
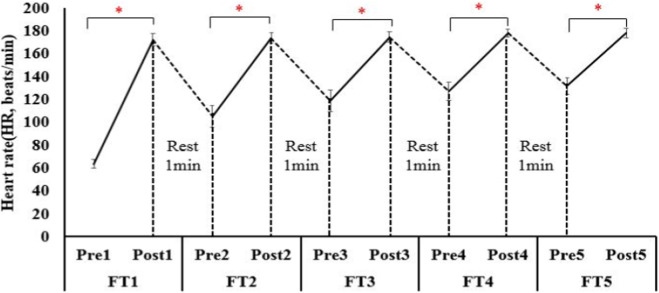
Changes of heart rate at pre-test and post-test of each FT. *, significant differences in each FT between pre-test and post-test (P < 0.001).

**FIGURE 5:**
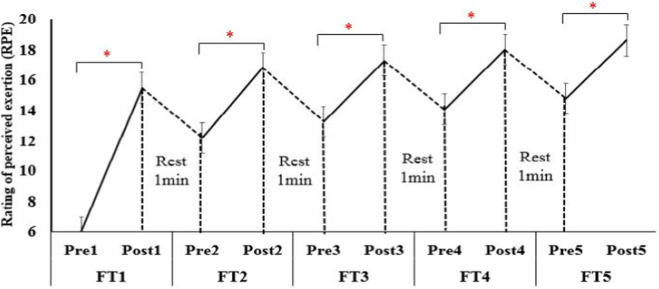
Changes of RPE at pre-test and post-test of each FT. *, significant differences in each FT between pre-test and post-test (P < 0.001).

**FIGURE 6:**
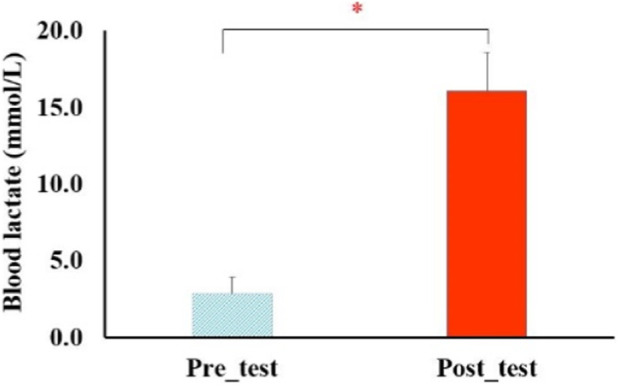
Changes of BL in pre-test and post-test. *, significant differences between pre-test and post-test (P < 0.001).

Heart rate increased from 63.5 ± 3.8 bpm at baseline to 178.0 ± 3.9 bpm after set 5 (F_4, 196_ = 1245.8, p < 0.001, η^2^p = 0.96, large effect size; Figure 4), reaching 92% HRmax—identical to the termination criterion used in the protocol by Ooi et al. (2023) (Enoka and Duchateau, 2008). RPE increased monotonically from 6 (no exertion) to 18.7 ± 0.9 (near-maximal) after the final set (F_4, 196_ = 1018.3, p < 0.001, η^2^p = 0.95, large effect size; Figure 5). BL concentration increased from 2.82 ± 1.12 mmol.L^-1^ pre-test to 16.07 ± 2.52 mmol.L ^-1^ post-test (t_4, 9_ = 31.9, p < 0.001, Cohens’ d = 6.8, large effect size; Figure 6), exceeding the lactate threshold of 8–10 mmol.L^-1^ typically reported for high-intensity intermittent racquet sports (Girard and Brocherie, 2022).

The total distance covered decreased from 273.40 ± 85.99 m in set 1 to 192.05 ± 91.40 m in set five (p < 0.001), with mean running velocity remaining stable across sets (approximately 2.79 m s^-1^, p = 0.41; Table 2). These kinematic patterns reflect the pacing strategies observed in collegiate badminton players during simulated match-play (Claudino, 2023a), supporting the ecological validity of the FT.

**TABLE 2:** Running characteristic of 15 male non-elite badminton players each FT in the study ^a^.

Parameters	FT1	FT2	FT3	FT4	FT5
Set of time, s	97.71 ± 29.74	74.18 ± 25.93*	71.12 ± 22.08*	65.73 ± 23.87*	69.57 ± 32.91*
Distance, m	273.40 ± 85.99	204.09 ± 67.87*	198.30 ± 64.63*	181.94 ± 64.01*	192.05 ± 91.40*
Speed, m/s	2.79 ± 0.22	2.79 ± 0.40	2.80 ± 0.43	2.79 ± 0.34	2.77 ± 0.53

^a^ Mean ± SD; *, significant differences from FT1, p < 0.001; RPE, rating perceived exertion; HR, heart rate.

In summary, the large effect sizes for VJ (η^2^p = 0.60), HR (η^2^p = 0.96), and RPE (η^2^p = 0.95) all exceeded the threshold for a large effect (η^2^p ≥ 0.14) as proposed by Brydges (Brydges, 2019), indicating that the modified FT elicited substantial peripheral fatigue responses in non-elite male players.

## 4 Discussion

Badminton is an intermittent high-intensity sport that repeatedly challenges the neuromuscular system of the lower limbs. Although exercise-induced peripheral fatigue has been widely studied in elite athletes, its manifestation and extent in non-elite athletes remain unclear, partly due to the lack of sport-specific field testing protocols. To address this issue, we developed a modified badminton field test (FT) and examined its ability to induce and quantify peripheral fatigue, in addition to its safety, in non-elite male college players. The findings of the present study demonstrate that a single 15-min modified badminton field test (FT) robustly induced peripheral fatigue in non-elite male collegiate players. We observed a 39.9% reduction in VJ height, a rise in BL to 16.1 mmol L^-1^, and attainment of 92% HRmax and near-maximal RPE. The changes in these fatigue markers were all associated with large effect sizes. The convergent metabolic, neuromuscular and perceptual changes confirm the efficacy of the protocol and provide an ecologically valid tool for quantifying sport-specific fatigue in this under-researched population. Furthermore, the completion of all five sets by all 15 participants without any injuries or adverse events indicates a high level of safety for the FT when applied to non-elite players.

The observed decline in VJ aligns with the 35%–45% reductions reported by Ooi and Tan (2023) after a simulated singles match in elite players and exceeds the 20%–30% decreases typically seen in repeated-sprint protocols for soccer (Girard and Brocherie, 2022). BL concentrations (≈16 mmol L^-1^) are consistent with values documented in official badminton tournaments (Phomsoupha and Laffaye, 2024b; Ooi and Tan, 2023) but higher than those following the Yo-Yo IR1 test (10–12 mmol L^-1^) (Castagna and Impellizzeri, 2021), underscoring the greater glycolytic demand of the FT. Similarly, the near-maximal RPE (18.7) concurs with recent data from collegiate tennis players (Gomes, 2022), reinforcing the external validity of our protocol. Unlike previous laboratory studies involving isokinetic dynamometry (Claudino, 2023b), the present FT integrated on-court movement patterns, thereby extending the generalizability of fatigue monitoring to resource-limited settings.

Peripheral fatigue in this study appears to be governed by two synergistic mechanisms. First, the marked lactate accumulation elevated extracellular [H^+^], which in turn reduced Ca^2+^ sensitivity and impaired actin–myosin cross-bridge cycling (Cheng and Place, 2021). Second, the progressive VJ decline suggests compromised excitation–contraction coupling, consistent with the results reported in the 2023 review by Girard and Brocherie (2022). From a practical standpoint, the FT offers coaches a 15-min, low-cost, and high-safety protocol to identify when players have reached critical fatigue thresholds. Integrating FT outcomes with session-RPE could inform real-time training-load adjustments, potentially reducing injury incidence attributed to peripheral fatigue in collegiate badminton athletes. By measuring the subjects’ exercise performance, physiological responses, and subjective feelings of fatigue following the FT protocol, we can not only assess exercise-induced fatigue but also contribute to monitoring training loads.

Study strengths include (i) the incorporation of sport-specific movement patterns, (ii) the concurrent assessment of metabolic, neuromuscular, and perceptual indicators, and (iii) the use of a homogeneous sample of non-elite athletes, which enhances ecological validity. Limitations include the non-randomized controlled trial design, the relatively small sample size and absence of female participants, and the lack of central fatigue measurements (e.g., maximal voluntary contraction with twitch interpolation). However, due to individual differences, it was not possible to rank individuals based on their fatigue levels with the FT, nor could we determine the weights of fatigue evaluation indicators, including athletic performance, physiological responses, biochemical changes, and subjective fatigue perception. Additionally, future studies should extend the FT to female and youth cohorts, and incorporate central fatigue measures to refine diagnostic precision.

## 5 Conclusion

This study provides the first empirical evidence that a 15-min, low-cost, sport-specific field test reliably induces and quantifies marked peripheral fatigue in non-elite male badminton players. The convergent findings—a 39.9% decline in vertical jump height, blood lactate accumulation to 16 mmol L^-1^, and attainment of 92% HRmax—collectively validate the protocol against elite match data. By incorporating authentic on-court movement patterns, the modified FT offers coaches and clinicians an ecologically sound and time-efficient tool to monitor training load and to intervene before fatigue-related injuries occur. Future research should (i) validate the FT in female and youth cohorts, (ii) integrate longitudinal monitoring to establish causal links between training load and fatigue, and (iii) incorporate central-fatigue indices to refine its diagnostic precision.

## Data Availability

The original contributions presented in the study are included in the article/supplementary material, further inquiries can be directed to the corresponding author.
